# 

**Published:** 1830

**Authors:** 


### ESSAYS AND CASES.

ART. PAGE.

I. Observations on the Pathology of Fever, with Strictures on the Physiological Doctrine of Broussais. By John Eberle, M. D., Professor of Materia Medica and Obstetrics, in the Jefferson Medical College, - - - 9

II. An unsuccessful case of Cynanche Trachealis, in which Tracheotomy was resorted to. An extract of a letter to E. Atlee, M. D., from John L. Atlee, M. D., of Lancaster. 23

III. Report of a case resembling Dry Gangrene, in which amputation of the thigh was performed. By William Lindsey. - - - - - - - 28

IV. Case of Chronic Dyssentery of three years duration, cured by the blue pill, and a mucilaginous diet. By Dr. R. Bard, M. D., of N. Troy, Vermont. - - - 31

V. A case of successful employment of the Rhus Toxicodendron in paralysis. By John Eberle, &c. &c. - 33

VI. A description of a disease called Charbon, that prevails between the Sabine and Red Rivers, communicated in a letter to Dr. Sparks, from James Z. Ellison, M. D., of Opalousas. - - - - - - - 35

VII. Cases illustrating the pathology of what are called "Cold Cases" of Bilious Fever. By James C. Finley, M. D. 39

VIII. History of two cases of Burn producing serious constitutional irritation. By Daniel Drake, M. D. - 48

IX. A. sketch of the life and character of John Armstrong, M. D. 60

### REVIEWS AND BIBLIOGRAPHICAL NOTICES.

X. An Essay on the remittent and intermittent diseases, including, generally Marsh Fever and Neuralgia. Comprising, under the former, many anomalies, obscurities, and consequences, and under a new systematic view of the latter, treating of Tic Douloureux, Sciatica, Headach, Ophthalmia, Toothach, Palsy, and many other modes and consequences of this generically disease. By John Macculoch, M. D., F. R. S., etc., etc. Physician in ordinary to his Royal Highness Prince Leopold of Saxe Cobourg. - - - - - - - - - 67

### MISCELLANEOUS INTELLIGENCE.

### I. Analectic.

1. Nerves of the Placenta. 2. Urachus. 105—3. State of the uterus three years after the Cœsarean operation. 4. Lymphatics and veins communicate with each other. 5. Abnormal Menstruation. 106.—6. Wedemeyer on the circulation. 107—7. Case in which no division existed between the ventricles the heart. 109.—110—10. Congenital absence of the Iris without loss of Vision. 111.—11. On the Regeneration of Bone. 112.—12. Instance in which life was supported for a length of time by the absorption of the fluids of the body. 13. Loss of sensation in one-half the body, without loss of the power of motion. 113—14. Intermittent Aphonia of thirty years standing. 114—16. Paralysis of the muscles of the face from disease of the Portio Dura. 116—17. Dupruyten's method of removing fibrous polypi of the Uterus. 117—18. Extirpation of the Uterus. 119—19. Croup in an adult. 20. Wax as an application to Ulcers. 21. Tinea Capitis. 121.—22. Case of fracture and depression of the inner table of the scull. 23. Dislocation of the clavicle forwards. 122.—24. Nicotin, the active principle of Tobacco. 123.—25. Jalap the product of an apomœa. 26. Strychnia in paralysis. 124.—Strychnia in chronic diarrhea. 131.—27. Cataracts altering with Diabetes. 132.—28. Infanticide. 134.

### II. Analytical.

1. Excision of the elbow joint. 2. Case of Carotid Aneurism. 183—.2 Warm bath in Delirium Tremens. 140.—5. Observations on Hematosis. 144.—6. Extraordinary case of pregnancy in which the fœtus was discharged through the abdominal viscera. 150.—7. Extirpation of the inverted Uterus. 152.

### III. Original.

1. Case of Obstetrics. 154.—2. Plurality of fœtuses in the mare. 155.—3. Dry Gangrene. 156.—4. Unusual Distribution of the Arteries. 157.—Obituary—Jefferson Medical College. 158.—Medical Graduates.—Publisher's Notice. 160.

---

### NO. II.

### ESSAYS AND CASES.

Art. Page.

I. Some Remarks on the Pathology and Treatment of Dysmeorrhea. By John. Eberle, M. D. - - - - 161

II. Remarks on Vaccination; on its value as a preventative of Small Pox, and the causes of its occasional failure. By James. C. Finley, M. D. - - - - - - 166

III. Remarks on Iodine, read before the Medical and Philosophical Society of Cincinnati. By Landon C. Rives, M. D. 176

IV. Case of Conical Cornea, communicated. By James M. Staughton M. D., late professor of Surgery in the Medical Department of the Columbian College, now of Cincinnati. - - - - - - - - - 188
V. A Case of Severe Nervous Irritation, from the sting of the insect called Yellow Jacket, [*Vespa Crabro*,] communicated in a letter from Dr. S. Littel, Jr., to Dr. Drake. - 192
VI. A Case of Rupture of the Bladder, terminating fatally on the 4th day. By John E. Bush, M. D., of Cincinnati - 194
VII. History of a Case of Exophthalmos or Protruded Eye-ball. By Daniel Drake, M. D. - - - - - - - 197

### REVIEWS AND BIBLIOGRAPHICAL NOTICES.

VIII. An Essay on the Remittent and Intermittent Diseases including Generically, Marsh Fever, and Neuralgia, &c. &c. By James M'Culloch, M. D., &c. - - - 213

### MISCELLANEOUS INTELLIGENCE.

### I. Analectic.

1. Some Symptoms in Children, erroneously attributed to Congestion. 257.—2. On the inefficacy of Ammonia, and the efficacy of the Cold Effusion, in the treatment of Poisoning with Hydrocyanic Acid. 263.—3. On the Evidence of General Poisoning. 265.—4. On the Diagnosis of Hernia. 273.—5. Excision of the Nerves in cases of Neuralgia. 275.—6. Cæsarian operation on a patient dead from Hæmoptysis. 277.—7. Diagnosis of Gastric and Intestinal Inflammation, by inspection of the features of the face. 279.—8. Lupus treated by the Iodide of Mercury. 280.—9. Case of Cystocele, which was not recognized for many years. 281.—10. The Lower Orifice of the Nasal Canal. 282.—11. Spontaneous Combustion. 283.—12. Hydrocephalus. 284.—13. Salicia in the Treatment of Intermittent Fever. 285.—14. Treatment of Puerperal Mania. 286.—15. Excision of the Knee Joint. 287.—16. Premature Labor. 287.—17. Preparation of the Chloride of Magnesium. 288.

### II. Analytical.

1. Gerhardon the Endermic Administration of Medicine. 289.—2. Report on the Trial of Jonathan Martin, for setting fire to the York Minster. 296.—3. Rutgers Medical College; An Introductory Lecture delivered at the College of Physicians in the city of New-York. 306—Jefferson Medical College. 311.

### NO. III.

### ESSAYS AND CASES.

ART. PAGE.

I. Sketch of the Life of Ambrose Parey. By J. M. Staughton, M. D. - - - - - - - 313

II. Case of Fracture of the Occipital Bone, with Lesion of the Brain, successfully treated. By John Ament Haldeman, M. D., of Fayette, Missouri. - - - - - 328

III. Observations on the Final Cause of Menstruation. By James C. Finley, M. D. - - - - - - 332

IV. Case of Epilepsy, communicated by J. M. Staughton, M. D. 340

V. Remarks on the Influence of Acids in promoting Salivation, communicated in a letter to Dr. Drake, by Thomas Townsend, M. D. - - - - - - - - - 343

### REVIEWS AND BIBLIOGRAPHICAL NOTICES.

VI. A. Practice of Physic, comprising most of the Diseases not treated of in "Diseases of Females," and "Diseases of Children." By W. P. Dewees, M. D., in two volumes. Philadelphia, Carey & Lea, 1830. pp. 600. - - 348

VII. Principles of Military Surgery, comprising Observations on the Arrangement, Police, and Practice of hospitals, and on the History, Treatment, and Anomalies of Variola and Syphilis; illustrated with cases and dissections. By John Hennen, M. D. F. R. S. E. - - - - - 376

VIII. The Influence of Modern Physical Education of Females, in producing and confirming diseases of the Spine. By E. M. Duffin, Surgeon. - - - - - - - 404

### MISCELLANEOUS INTELLIGENCE.

### I. ANALECTIC.

1. Incontinence of Urine cured by an Operation. 415.—2. Recovery from Ruptured Uterus. 416.—3. Alteration of the Blood in Yellow Fever. 417.—4. A Strychnine in Amaurosis. 418.—5. Gonorrhœa alternating with Bilious Remittent Fever. 420.—6. Mysterious Expectoration of Pus. 421.—7. Experiments on the coloring of different Tissues. 422.—8. Hernia of the Stomach with enormous enlargement of that organ. 423.—9. Phlegmasia Dolens in a Male. 10. On the Morbid Phenomena caused by Iodine. 424.—11. Clinical Reports of the Effects of Venesection in the cold stagas of Intermittent Fever. 425.—12. On the Treatment of certain Injuries of the Eye. 426.—13. Amaurosis. 428.—14. Portion of bone lodged for forty-eight days in the Trachea of an Infant.—15. Strabismus. 429.—16. Protracted utero-Gestation.—17. Poisoning by Opium and Belladonna used as an Injection. 430.—18. Notice of Iron found in the Cinchona Bark. 431.—19. On Aromatic or Spiced Rhubarb.

432.—20. Formulæ. 433.—21. Effect of light on plants. 434.—22. Remarkable reunion of a divided part.—23. Chorea. 24. Mr. Wardrops' method of treating Nævus. 435.—25. Evacuation of the Humours of the Eye by a wound, with recovery of sight. 436.—26. Tetanus caused by a foreign body embedded in the Ulnar Nerve.—27. Dupuytren's treatment of Hernia Humoralis. 438—28. Excision of the Upper Jaw. 439.—29. Tapping in Hydrocephalus. 440.—30. Case of Recovery from a wound in the stomach inflicted by a pistol ball. 441.—31. The influence of Electricity on Animal Putrefaction. 442.—32. Purity of Balsam Copaiba.—33. Transfusion. 443.—34. New Pessary. 35. The Use of the Acetate of Lead in Ulcerated Phthisis. 36. Fracture of the Spine. 36. Seat of Taste. 444.—37. Case of Illustrating the intoxicating effects of Sulp. of Quina. 38. Distinctive marks of Pregnancy. 446.—39. Artificial eyes. 449.—40. Mode in which the Poison of Lead is conveyed into the system. 41. Division of the Submaxillary and other Nerves. 450.—42. Case of Labor in which the placenta was delivered some time before the Foetus. 452.—43. Peculiar kind of Traumatic Delirium. 453.—44. Biographical Notice of Soemmering. 456.—45. Obituary of Dr. Wells. 458.

### II. Original.

Lithontrity. Dr. A. G. Smith. 461.

---

### NO. IV.

### ESSAYS AND CASES.

Art. Page

I. An account of the Extirpation of the Parotid Gland. In a letter from Professor M'Clellan to the Editors of the Western Journal. - - - - - - - - 465

II. Account of an Epidemic Bilious Pleurisy, which occurred in Appling, Columbia Co., Georgia, in the fall and winter of 1830. By Dr. Allen Kimball. - - - - 473

III. A Case of successful operation for the Excision of the Ossified Cartilages and anterior extremities of two Carious Ribs, and the lower portion of the Sternum. By Geo. M'Clellan, M. D., Professor of Surgery in Jefferson College. - - - - - - - - - - 479

IV. Report of a Case terminating fatally from the local application of Corrosive Sublimate in the cure of a species of Tetter. (Herpes exedens. By Dr. Allen Kimball, of Appling, Columbia Co., Georgia. - - - - - - 483

V. Notice of the Hooping Cough and Measles appearing together in the same patient, as it occured at Newport and its vicinity, Herkimer Co. N. Y. By A. B. Bowen, M. D. 485

VI. Removal of a large Steatomatous Tumor from the Neck. By John C. Brent, M. D., of Shelbyville, Ky. - - 487

VII. Case of Pulmonary disease threatening Phthisis, relieved by the supervention of Colica Pictonum, in consequence of drinking cider impregnated with lead. By John E. Bush, M. D., of Cincinnati. - - - - - - 489
VIII. Remarks on the Influence of Hysteria, in modifying the Symptoms of Febrile Diseases. By James C. Finley, M. D. - - - - - - - - 492

### REVIEWS AND BIBLIOGRAPICAL NOTICES.

IX. Manuel Pratique de la Lithotritie, ou Lettres a un Jeune Medecin, sur le traitement de la Pierre dans la Vessie. Par A. P. Bancal, Medecin a Bordeaux, Paris.
Practical Manuel of Lithrotrity, or Letters to a young Physician on breaking down the Stone in the Bladder. - - 496
X. The French Practice of Medicine: being a translation of L. J. Begin's Treaties on Therapeutics, &c., &c. By Xavier Tessier. - - - - - - - 518
XI. An Essay on Venereal Diseases, and the uses and abuses of Mercury in their treatment. Illustrated by drawings of the different forms of Venereal Eruptions. By Richard Carmichael. With Practical Notes, &c., by G. Emerson, M. D., of Philadelphia: Hennen's Observations on Syphilis - - - - - - - - - 545

### MISCELLANEOUS INTELLIGENCE.

### I. Analectic.

1. Case of Amnesia. 569.—2. Berard on the Veins. 571.—3. Catalepsy. 572.—4. Efficacy of Cold Effusions in Nervous Affections. 575.—5. Homicidal Monomania. 576.—6. Inflammation of the Vena Cava Ascendens. 580.—7. Chlorine an Antidote to Hydrocyanic Acid. 581.—8. Excision of a Carious Rib. 582.—9. Method of Cleaning Bones. 583.—10. Period of Puberty in women. 583.—11. Menstruation continued to the 94th year. 584.—12. Pregnancy occurring during Dysmenorrhea. 584.—13. Salicine not an alkali.—14. Wound of the Trachea and Œsophagus. 585.—15. Strangulation of the Spermatic Cord in the Abdominal Canal. 586.—17. Ununited Fracture of the Femur successfully treated by Seton. 588.—18. Removal of the Penis by Ligature. 589.—19. Poisoning by Mouldy Bread. 590.—20. Case of Spasmodic Asthma relieved by Inhalation of Ether, Conium, and Ipecacuanha. 590.—21. Test for distinguishing different kinds of Rhubarb. 591.—22. New Operation for Ectropion. 591.—23. Opacities of the Cornea.—591.—24. Cure of Subcutaneous Nœvus by the Seton. 592.—25. On Lithoplatomy. 592.—26. Camphor. 595.—27. Economic Lightning. 596.

### II. Original.

28. Biographia Medica. 596.

### CONTENTS OF NO. 1, VOL. IV---JULY, 1830.

Art. I. Observations on the Pathology of Fever, with Strictures on the Physiological Doctrine of Broussais. By John Eberle, M. D., Professor of Materia Medica and Obstetrics in the Jefferson Medical College, - - - - - 9

II. An unsuccessful case of Cynanche Trachealis in which Tracheotomy was resorted to. An extract from a letter to E. Atlee, M. D., from John L. Atlee, M. D., of Lancaster, 23

III. Report of a case resembling Dry Gangrene, in which amputation of the thigh was performed. By William Lindsey, 28

IV. Case of Chronic Dysentery of three years duration, cured by the blue pill and a mucilaginous diet. By D. R. Bard, M. D., of N. Troy, Vermont, - - - - - - - 31

V. A case of the successful employment of the Rhus Toxicodendron in paralysis. By John Eberle, M. D., &c. &c. 33

VI. A description of a disease called Charbon that prevails between the Sabine and Red Rivers, communicated in a letter to Dr. Sparks, from James Z. Ellison, M. D. of Opolousas 35

VII. Cases illustrating the pathology of what are called "Cold cases" of Bilious Fever. By James C. Finley, M. D. 39

VIII. History of two cases of Burn producing serious constitutional irritation. By Daniel Drake, M. D. - - 48

IX. A sketch of the Life and Character of John Armstrong, M. D. - - - - - - - - - - - 60

### REVIEWS AND BIBLIOGRAPHICAL NOTICES.

X. An Essay on the remittent and intermittent diseases, including, generally Marsh Fever and Neuralgia. Comprising, under the former, various anomalies, obscurities, and consequences, and, under a new systematic view of the latter, treating of Tic Douloureux, Sciatica, Headach, Ophthalmia, Toothach, Palsy, and many other modes and consequences of this generic disease. By John Macculloch, M. D., F. R. S., etc. etc. Physician in ordinary to his Royal Highness Prince Leopold of Saxe Cobourg - - - - 67

### MISCELLANEOUS INTELLIGENCE.

#### I. Analectic.

1. Nerves of the Placenta. 2. Urachus, 105—3. State of the Uterus three years after the Cœsarean operation. 4. Lymphatics and veins communicate with each other. 5. Abnormal Menstruation, 106.—6. Wedemeyer on the circulation, 107.—7. Case in which no division existed between the ventricles of the heart, 109.—9. Influence of the male extending beyond a single impregnation, 110.—10. Congenital absence of the Iris without loss of Vision, 111.—11. On the Regeneration of Bone, 112.—12. Instance in which life was supported for a length of time, by the absorption of the fluids of the body. 13. Loss of sensation in one-half the body, without loss of the power of motion, 113.—14. Intermittent Aphonia, 114.—15. Intermittent Aphonia of thirty years standing, 115.—16. Paralysis of the muscles of the face from disease of the Portio Dura, 116.—17. Dupuytrens method of removing fibrous polypi of the Uterus, 117.—18. Extirpation of the Uterus, 119.—19. Croup in an adult. 20. Wax as an application to Ulcers. 21. Tinea Capitis, 121.—22. Case of Fracture and depression of the inner table of the skull. 23. Dislocation of the clavicle forwards, 122.—24. Nicotin, the active principle of Tobacco, 123.—25. Jalap the product of an apomœa. 26. Strychnia in paralysis, 124.—Strychnia in chronic diarrhea, 131.—27. Cataracts alterating with diabetes, 132.—28. Infanticide, 134.

### II. Analytical.

1. Excision of the elbow joint. 2. Case of Carotid Aneurism, 138.—3. Warmbath in Delirium Tremens, 140.—5. Observations on Hematosis, 144.—6. Extraordinary case of pregnancy in which the fœtus was discharged through the abdominal viscera, 150.—7. Extirpation of the Inverted Uterus, 152.

### III. Original.

1. Case of Obstetrics, 154.—2. Plurality of Fœtuses in the mare, 155.—3. Dry Gangrene, 156.—4. Unusual Distribution of the Arteries, 157.—Obituary—Jefferson Medical College, 158.—Medical Graduates,—Publishers Notice, 160.

